# Hepatitis-C and it's seroconversion in end stage kidney disease patients on maintenance hemodialysis and factors affecting it

**DOI:** 10.12669/pjms.35.1.366

**Published:** 2019

**Authors:** Yasir Hussain, Anjum Shahzad, Sidra Azam, Nazish Munawar

**Affiliations:** 1 Dr. Yasir Hussain, FCPS (Nephrology). Department of Nephrology, DHQ hospital, Sheikhupura, Pakistan; 2 Dr. Anjum Shazad, FCPS (Medicine). Department of Nephrology, PGMI/ Lahore General Hospital, Lahore, Pakistan; 3 Dr. Sidra Azam (House Physician). Department of Nephrology, PGMI/ Lahore General Hospital, Lahore, Pakistan; 4 Mrs. Nazish Munawar (Pharmacist). Department of Nephrology, DHQ hospital, Sheikhupura, Pakistan

**Keywords:** Seroconversion, Hepatitis-C, Hemodialysis

## Abstract

**Objective:**

To determine frequency of hepatitis-C in dialysis patients at start of hemodialysis, seroconversion from HCV negative to HCV positive over study duration and factors affecting seroconversion.

**Methods:**

This descriptive prospective observational study done in dialysis center of DHQ hospital Sheikhupura, Punjab, Pakistan. The study was conducted from October 2016 to October 2017. Data was collected on Performa and later followed prospectively in same cohort of patients. All the patients on maintenance hemodialysis for more than one month were included in the study. Patients with acute kidney disease and on dialysis less than one month were excluded. Patients were analyzed by dividing them in three groups, group-I patients who were HCV positive at start of dialysis, Group-II who were negative and seroconvert to HCV positive, Group-III who were negative and remained negative. All seronegative patients were followed at one, three, six and twelfth months on being hemodialysis for seroconversion.

**Results:**

Out of 230 surveyed patients 52 were HCV positive at start of dialysis and 19 were loss of follow up. Out of remaining 159 HCV negative patients 95 became HCV positive, only 64 patients remained HCV negative by end of study.

**Conclusion:**

Frequency of HCV seroconversion among chronic hemodialysis patient is found to be 53.37%. Arteriovenous access, number of dialysis, reuse of dialyzer and blood transfusions are important risk factors.

## INTRODUCTION

Chronic Kidney Disease (CKD) may leads to End Stage Kidney Disease (ESKD) which itself is a big burden for health and finance.^1^ Prevalence of CKD in Pakistan ranges from 12.5-22.6%.^2^ It’s incidence is progressively increasing in Pakistan due to multiple factors like inadequate health care services, health education is minimal, inadequate funding in health department by government, excessively prevalent risk factors of disease like diabetes and hypertension in general population and dry weather conditions favoring glomerulonephritis and renal stones.^3^ Hemodialysis (HD) in centers is major modality of treatment for ESKD in Pakistan as chronic Peritoneal Dialysis (PD) is more expensive and nephrologist dependent.^4^

Hepatitis-C virus (HCV) is an RNA virus member of the family Flaviviridae. Global prevalence of HCV is 2.5%.^5^ Seroprevalence in general population of Pakistan is 6.8%.^6^ The prevalence of HCV among HD patients varies greatly by geographic area (4% to 59% in different countries) throughout the world. Patients on hemodialysis are at high risk of seroconversion (appearance of anti-HCV antibody after exposure), like thalassemia and ICU patients, because of multiple reasons, important being are multiple blood transfusions, frequent needling, blood sampling and extracorporeal circulation and decreased cellular immunity^7^,^8^ Seroconversion to hepatitis-C (HCV) in patients on HD is as low as 1.1% in UK to as high as 48.9% in Pakistan.^9^,^10^ Although hepatitis-C infection has slow progressive course but in this population course seemed to be atypical because of delayed Seroconversion secondary to poor nutrition and immunity. These patients present very late because of late seroconversion and most of them are seronegative. Most common way of detection in dialysis center is by ELISA (enzyme linked immunosorbent assay) method which usually misses seronegative PCR positive patients.^11^

## METHODS

A simple Observational study was conducted at Dialysis center of DHQ hospital, Sheikhupura, Punjab, Pakistan. All patients with ESKD on maintenance dialysis for more than one month, were included in study and followed up for next twelve months i.e. October 2017. A total of 230 patients 16 to 75 years old patients were in our study group 19 were excluded due to loss of follow up or change of center. Patients with Acute kidney injury (AKI), Patients with ESKD who came to center for short time period (less than one month), Co-infection with HIV or hepatitis B and Patients with age <15 or >75 were excluded from study. Data of all patients fulfilling the study criteria were enrolled in the study. Demographic data of the patient’s (age, gender, occupation and marital state) was collected by direct interview, duration on HD, history of blood transfusion(s), attending more than one HD center and type of vascular access for HD (arterio-venous fistula, double lumen catheter, permanent catheter) number of blood transfusions per month was taken from record and noted in Performa. Serological results of patients were acquired from center records and compared with current serological status and only seronegative patients were followed for their serology status at one, three, six and twelve months. All data was put in SPSS-23 and analyzed for rate of seroconversion.

### Statistical Analysis

Data were stored and analyzed using SPSS-IBM version 23.0, count and percentages were reported for qualitative variables like age group, gender, education, employment etc, mean and standard deviation was given for quantitative biochemical parameters, Pearson chi square test was done to see the association of viral infection with baseline and clinical parameters, independent sample t-test was used to compare the mean of biochemical parameters, binary logistic regression was done to estimate the odds with 95% confidence interval for HCV positive, p-values less than 0.05 were considered significant. Flow chart was also used to present the count and percentages of studied samples.

**Figure:**
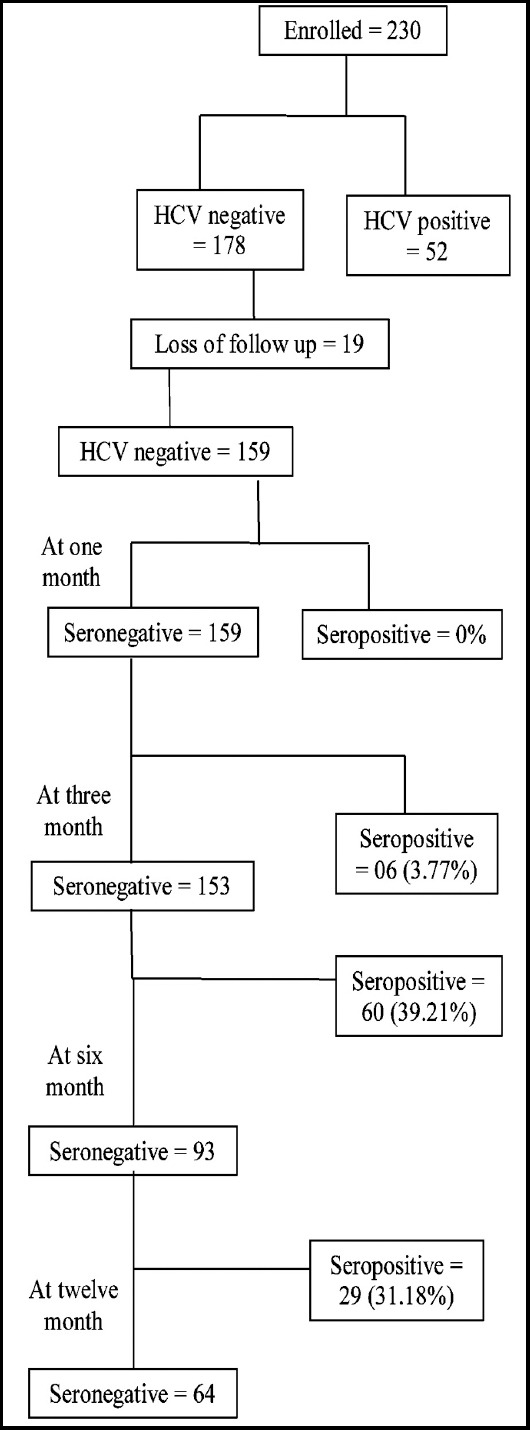
Flow sheet showing seroconversion at 1, 3, 6 and 12 months of study.

## RESULTS

Total 230 patients were enrolled in this study. Nineteen were excluded due to loss of follow up or change of center. All patients were on maintenance dialysis, two sessions a week, each session of four hours, seropositive patients were being dialyzed on same floor but on separate dedicated machines. One dialyzer was being reused thrice for dialysis. Chemical disinfection was done after each dialysis. Majority (69.17%) was aged above 40 years. One hundred and twenty two were male. Most (78.67%) were unemployed, 53.08% were living in rural areas of district Sheikhupura. Major causes of ESRD were diabetes mellitus (DM) 46.92%, hypertension (HTN) 41.70%, chronic glomerulonephritis (GN) 3.81%, and cystic disease 2.84%, other causes were in 2.84% of studied population. Most of patients 83.88% started dialysis with double lumen catheter only 16.12% were dialyzed via arteriovenous fistula (AVF). Almost all patients (99%) are being dialyzed twice weekly. Only 28.92% of patients got blood transfusions 2-4 times a month and 9.00% had transfusion for more than four times a month. All patients were receiving erythropoietin 4000 IU twice weekly. Out of the 230 surveyed patients, 178 were negative for HCV and 52 were positive at time of enrollment; 19 were loss of follow up, the frequency of HCV infection at start of study was 22.2%. Out of these 159 HCV negative patients, 95 became seropositive for HCV. Six were seroconverted at three months, sixty at 6 months, twenty nine at one year. So sero conversion was seen in 95 out of 159 (59.74%).

Table-I shows the baseline characteristics, of 211 samples, there was no significant association observed of baseline parameters with viral infection. Table-II shows association of viral status with dialysis and other risk parameters.

**Table-I:** Baseline Characteristics of studied Samples (n=211) at start of dialysis

Characteristics		Number (%)	p-value
Age group	≤40 Years	65 (30.81%)	0.48
	>40 Years	146 (69.19%)	
Gender	Male	122 (57.82 %)	0.35
	Female	89 (42.18 %)	
Education	Literate	121 (57.82 %)	0.36
	Illiterate	90 (42.65 %)	
Employment status	Employed	45 (21.33 %)	0.72
	Unemployed	166 (78.67%)	
Address	Urban	99 (46.92 %)	0.40
	Rural	112 (53.08 %)	
Cause of ESKD	DM	99 (46.92 %)	0.76
	HTN	88 (41.70 %)	
	Stone disease	4 (1.89 %)	
	chronic GN	8 (3.81 %)	
	cystic disease	6 (2.84%)	
	Other	6 (2.84 %)	

*p<0.05 was considered significant using Pearson Chi Square test.

**Table-II:** Association of Viral Status with Dialysis and other risk parameters.

Characteristics		Number (%)	p-value
Dialysis start via	AVF	34 (16.12 %)	<0.01*
	double lumen catheter	177 (83.88 %)	
Total number of dialysis	<50	49 (23.22 %)	<0.01*
	50-100	53 (25.11 %)	
	>100	109 (51.66 %)	
Need for transfusion	Yes	159 (75.36 %)	0.156
	No	52 (24.64 %)	
Blood transfusion required	<2 times per month	131 (62.08 %)	0.075
	2-4 times per month	61 (28.92 %)	
	more than 4 times per month	19 (9.00 %)	

* p<0.05 was considered significant using Pearson Chi Square test

## DISCUSSION

Hepatitis-C infection is one of most prevalent infection in Pakistan. It can lead to Decompensated Liver Disease (DCLD) and even Hepatocellular Carcinoma (HCC).^12^ HCV is associated with increased risk of morbidity and mortality among patients on maintenance dialysis.^13^ A meta-analysis showed 1.57 times increased risk of death after HCV infection^14^ Prevalence of hepatitis-C in this study turned out to be 22.2%. Despite this high prevalence of HCV, seroconversion rate in our study was significantly higher in comparison with studies conducted in other countries like United Kingdom, USA, India, Egypt, Africa where SC rates are 1.2%, 2.5%^9^ 7.44%^15^ 14%^16^ 25%^17^ respectively. It is even higher than previous studies conducted in Pakistan proportioned to be 48.9%.^10^ Risk factors for high rate of seroconversion are considered to be multiple transfusions, chronic hemodialysis (HD), surgical procedures, multiple Injections, organ transplantation, occupational exposure among healthcare workers, unprotected sexual contact. Extensive use of recombinant erythropoietin for anemia correction has markedly reduced need for transfusions but still transfusion rate is high. Frequent transfusions are known risk factors for seroconversions. Majority of the patients (59.74%) of patients became positive within one year of start of dialysis and a study describes previously that early seroconversion is associated with blood transfusions.^18^ Studies done by Daniele P showed seropositivity rates in blood donors of Pakistan is up to 21%.^19^ Most of the patients (83.88%) started dialysis with temporary double lumen catheters; they had surgeries for AVF or graft for access for dialysis. Almost half (46.92%) patients had diabetes, some of them had its complications like multiple, recurrent abscesses, skin infections and had surgical procedures for that.

All HCV positive patients were being dialyzed on same floor in a restricted area on dedicated machines. Each dialyzer was being reused for three times after manual washing with acidic solution. This reuse might be source of infection but in previous studies dialyzer reuse was not considered as significant risk factor for seroconversion.^20^ There have been controversial reports in the previous studies regarding the importance of isolating HCV positive patients on HD.A literature review done by Huraib concluded that isolation was favorable.^21^ But isolating HCV-infected hemodialysis patients is not suggested by the Kidney Disease Improving Global Outcome (KDIGO).^22^

It has been suggested that infection could be transmitted from patient to patient in the hospital, and there is now indirect evidence that HCV infection occurs among HD patients during repeated dialysis procedures, but not through the equipment, probably due to procedural errors.

## CONCLUSION

Frequency of HCV seroconversion among chronic hemodialysis patient is found to be 53.37 %. Arteriovenous access, number of dialysis, reuse of dialyzer and blood transfusions are important risk factors. Universal precautions to control infection should be practiced in dialysis facility.

### Authors’ Contribution

**YH:** Conceived, designed and did statistical analysis & editing of manuscript.

**AS** **& SA:** Did data collection and manuscript writing.

**NM:** Did review and final approval of manuscript.
